# Effect of Heat Treatments under High Isostatic Pressure on the Transport Critical Current Density at 4.2 K and 20 K in Doped and Undoped MgB_2_ Wires

**DOI:** 10.3390/ma14185152

**Published:** 2021-09-08

**Authors:** Daniel Gajda, Andrzej J. Zaleski, Andrzej J. Morawski, Malgorzata Małecka, Konstantin Nenkov, Matt Rindfleisch, Md Shahriar A. Hossain, Tomasz Czujko

**Affiliations:** 1 Institute of Low Temperature and Structure Research PAS, Okolna 2, 50-422 Wroclaw, Poland; a.zaleski@intibs.pl (A.J.Z.); m.malecka@int.pan.wroc.pl (M.M.); 2 Institute of High Pressure Physics PAS, Sokolowska 29/37, 01-142 Warsaw, Poland; amor@unipress.waw.pl; 3 Institute for Solid State and Materials Research Dresden, P.O. Box 270016, D-01171 Dresden, Germany; nenkov1975@outlook.de; 4 Hyper Tech Research, Inc., 1275 Kinnear Road, Columbus, OH 43212, USA; mrindfleisch@hypertechresearch.com; 5 School of Mechanical and Mining Engineering, The University of Queensland, Brisbane, QLD 4072, Australia; md.hossain@uq.edu.au; 6 Institute of Materials Science and Engineering, Military University of Technology, Kaliskiego 2, 00-908 Warsaw, Poland

**Keywords:** MgB_2_ superconducting wires, high isostatic pressure, critical current density

## Abstract

Annealing undoped MgB_2_ wires under high isostatic pressure (HIP) increases transport critical current density (*J*_tc_) by 10% at 4.2 K in range magnetic fields from 4 T to 12 T and significantly increases *J*_tc_ by 25% in range magnetic fields from 2 T to 4 T and does not increase *J*_tc_ above 4 T at 20 K. Further research shows that a large amount of 10% SiC admixture and thermal treatment under a high isostatic pressure of 1 GPa significantly increases the *J*_tc_ by 40% at 4.2 K in magnetic fields above 6 T and reduces *J*_tc_ by one order at 20 K in MgB_2_ wires. Additionally, our research showed that heat treatment under high isostatic pressure is more evident in wires with smaller diameters, as it greatly increases the density of MgB_2_ material and the number of connections between grains compared to MgB_2_ wires with larger diameters, but only during the Mg solid-state reaction. In addition, our study indicates that smaller wire diameters and high isostatic pressure do not lead to a higher density of MgB_2_ material and more connections between grains during the liquid-state Mg reaction.

## 1. Introduction

MgB_2_ superconductors have many advantages in that they are cheap components [1] and have a low specific weight [2], low anisotropy [3], high critical temperature [1], low resistivity in the normal state [4], and high *B*_c2_ [5]. However, they have one large disadvantage: shrinkage during the synthesis reaction. The analysis of the MgB_2_ structure shows that the volume decreases by 25% after a reaction [6]. This in turn causes a reduction in the connections between grains and creates surface pinning centers that significantly decrease the critical current density (*J*_c_) in middle and high magnetic fields [7,8]. Doping, e.g., SiC and C, promotes middle and high pinning centers, which increase *J*_c_ in middle and high magnetic fields [7,9,10,11,12]. SiC doping is more effective for increasing *J*_c_ in high magnetic fields; however, it decreases the critical temperature (*T*_c_) [8,13,14] and increases the irreversibility magnetic fields (*B*_irr_) and upper magnetic fields (*B*_c2_) [8,10,15,16,17]. A 2002, an investigation of SiC showed that the addition of nanometer-scale SiC can effectively increase *B*_c2_ and transport the critical current density (*J*_ct_) at high temperatures and fields and decrease the anisotropy [11,13,18,19]. In contrast, large SiC grains lead to higher *J*_ct_ at low temperatures [19]. Additionally, the results showed that SiC nanoparticles with excess Mg improve *J*_c_ in low and high magnetic fields [14,18,20,21,22]. Furthermore, the examination showed that SiC doping increases the magnetic critical current density (*J*_cm_) in low and middle magnetic fields between 5 K and 25 K [9]. However, measurements indicated that SiC doping slightly increased *J*_cm_ in high magnetic fields [9]. Serrano et al. suggested that *J*_c_ is increased by strong pinning centers [23]. The measurements indicate that nano SiC increased *J*_c_ at 20 K more than other types of doping (e.g., carbon nanotubes) [23] and yielded a high irreversibility magnetic field (*B*_irr_) of 7.3 T [16]. An investigation conducted by Li et al. showed that *B*_irr_ is responsible for improved *J*_c_ performance in high magnetic fields [20]. Qu et al. suggested that impurities can increase or decrease *J*_c_ in MgB_2_ materials [16]. Moreover, Liang et al. indicated that the negative effects on *J*_c_ could be attributed to the absence of significant effective pinning centers (Mg_2_Si) due to the high chemical stability of crystalline SiC particles [24]. Moreover, more crystalline SiC nanoparticles located at the grain boundaries means more degradation of *J*_c_ [24]. The results suggest that the reaction of SiC with Mg might be a necessary condition for the enhancement of *J*_c_ in SiC-doped MgB_2_ wires [24]. Asthana et al. showed that ethyltoluene and SiC doping significantly increases *J*_c_ and *B*_c2_ in MgB_2_ tape more than ethyltoluene doping alone [25]. Additionally, measurements indicate that SiC-doped samples sintered at 650 °C have a better *J*_c_ than those sintered at 1000 °C [26]. The results for the SiC-doped MgB_2_ material showed that a lower heating time improves *J*_c_ while slightly degrading *T*_c_ [27].

An investigation performed by Shcherbakova et al. indicated that a long annealing time enhanced the connections between grains [27]. Further results showed that a higher density of MgB_2_ material led to better connections between grains [22]. Zhang et al. stated that leftover B constituted an impurity in MgB_2_ and degraded the connectivity [22]. The number of connections between grains is strongly related to the amount of impurities [20,22]. Moreover, the results indicated that Mg addition with 10% SiC increased the number of connections between grains [20]. However, Asthana et al. suggested that a smaller amount of MgO led to better connectivity of the MgB_2_ grains with SiC doping [25]. Serrano et al. showed that SiC doping weakly increased the number of connections between grains [23]. In addition, the results showed that SiC doping did not change the density of MgB_2_ material [13].

Jung et al. indicated that SiC nanoparticles react with Mg above 600 °C and form Mg_2_Si compounds [14]. Shcherbakova et al. showed that sintering a SiC-doped sample near the melting point of Mg (640–650 °C) resulted in large numbers of nanoprecipitates and grain boundaries [27]. Furthermore, the investigation showed that a longer cooling time created more Mg_2_Si impurities, producing more Mg vacancies [27]. Li et al. stated that liquid Mg first reacts with SiC to form Mg_2_Si and is then released free with B to form MgB_2_ and released free with Si at high sintering temperatures, leading to strong flux–pinning centers due to small particles being created [8]. The results showed that SiC doping created intragrain defects and a high density of nanoinclusions and impurities (Mg_2_Si, Mg_2_C_3_, and MgO unreacted SiC), which are effective pinning centers [9,13,21,28]. The results indicate that Mg_2_Si inclusions inside MgB_2_ grains significantly enhance the pinning strength in high magnetic fields [27]. Furthermore, the results showed that large impure particles such as unreacted SiC would not be effective pinning centers [21]. Dou et al. suggest that nanoscale precipitates with sizes below 10 nm (e.g., Mg_2_Si and BC) create strong pinning centers [21]. Shimada et al. showed that nano-SiC easily formed Mg_2_Si [27]. Moreover, analysis of the structure of MgB_2_ material made by using the internal Mg diffusion (IMD) method showed that large Mg_2_Si regions are distributed parallel to the drawing direction [29]. This may suggest that the SiC admixture will behave similarly in MgB_2_ wires made by the PIT method.

Research shows that both SiC and C doping reduces the a-axis parameter but does not change the c-axis parameter [10,15,30]. The reduction of the a-axis might cause Mg deficiency, oxygen occupancy on B sites, strain (pressure effect—0.5 GPa), and C doping on B sites [10]. However, Wang et al. stated that Mg vacancies cannot be the origin of a lattice reduction [10]. Furthermore, Dou et al. stated that, because the atomic radius of C is 0.077 nm, that of Si is 0.11 nm and that of B is 0.097 nm [13,28], carbon substitution for boron in the MgB_2_ lattice can lead to a large lattice distortion because of the shorter C-B bonds (1.71 A) [17,20,22,26]. Additionally, Kazakov et al. stated that the disorder should lead to a shortening of the Mg-C distance (in the range of 2.25–2.52 Å) [31]. Moreover, carbon substitution for boron leads to increased band scattering [17,20] and significantly enhances *B*_c2_ and *B*_irr_ [20]. Further research showed that the proportion of C-added SiC to substitute for B in the lattice is small compared to the pure C substitution case [21,27]. In addition, a small proportion of C substituted for B forms nano-domains in the MgB_2_ material [21]. These nanodomains are rectangular and approximately 2–4 nm [21]. Dou et al. stated that C substitution for B caused a reduction in the grain size [26]. Moreover, Serrano et al. suggested that C improved the connections between grains [23]. Others showed that C substitution for B in SiC-doped MgB_2_ led to an increase in the normal state resistivity [13,18,20,21]. This high resistivity might suggest that SiC doping creates poor connections between grains [22]. Kazakov et al. showed that C-substituted crystals decrease the coherence length [31]. C substitution for B occurs at temperature as low as 650 °C [26]. Dou et al. showed that a higher annealing temperature led to a higher level of C substitution than a low annealing temperature [26]. Moreover, Dou et al. and Kazakov et al. indicated that an increase in the C content in MgB_2_ material led to an increasingly larger reduction in *T*_c_ [26,31]. SiC and organic co-additions may increase the amount of C substitution for B [32]. Mg deficiency will increase as the carbon content increases [31].

Wang et al. suggested that strains are created by a chemical addition and are not influenced by the synthesis parameters because the reduction of the lattice is not observed in pure MgB_2_ material [10]. A longer annealing time decreases the lattice strain from 0.45 to 0.4% for pure MgB_2_ material [27]. In addition, the quenching of the samples creates more strains in the MgB_2_ lattice in both pure and SiC-doped MgB_2_ [27]. In contrast, long annealing durations in SiC-doped MgB_2_ create more strains (which are formed by Mg_2_Si) [27]. The strains lead to a number of crystal defects, such dislocations, which are strong pinning centers [27]. Transmission electron microscopy images showed that nano-SiC doping created a high density of dislocations, stacking faults, a large number of 10 nm-sized inclusions inside the grains [13,21,26,28], and inclusions and nanoscale impurities between grains, e.g., MgB_4_ and MgO [21]. Li et al. indicated that thermal strains originating from the interface of SiC and MgB_2_ are one of most effective sources of flux pinning centers to improve the supercurrent critical density [33]. Kazakov et al. indicated that local disorder introduced by carbon substitution increased the pinning force [31].

Previous results suggest that Si may also substitute into the crystalline lattice [13]. However, even a high pressure (30 kbar) and high annealing temperature (above 1900 °C) do not lead to a substitution of Si into the crystalline lattice [21]. Wang et al. and Ghorbani et al. also showed that Si cannot be incorporated into the crystal lattice [10,18].

Nanocrystalline SiC does not react with Mg [15,22,24] and is mostly located on the surface boundaries of MgB_2_ grains [24]. Unreactive crystalline SiC creates a high density of defects (dislocations and lattice distortion) in the structure of MgB_2_ material [15]. However, amorphous SiC reacts with Mg and creates nano-Mg_2_Si [15,24]. Amorphous SiC in MgB_2_ thick films creates strong pinning centers and improves intergrain connectivity [14]. Additionally, TEM images show strong bonding of MgB_2_ with crystalline SiC [15]. Zeng et al. showed that cooling MgB_2_ with unreactive crystalline SiC creates thermal strains, which are effective pinning centers [15]. Moreover, Zeng et al. indicated that strain effectively increases *J*_c_ [15]. Li et al. suggested that the combination of high connectivity and strong disorder led to high critical parameters [8,20]. Liang et al. suggested that, unlike crystalline SiC nanoparticles located inside MgB_2_ grains, crystalline SiC nanoparticles located at grain boundaries may not act as effective pinning centers [24].

Hot pressing increases the reaction rate between Mg and B [16]. Qu et al. showed that the density of the sample increases as the hot pressing temperature increases [16]. Moreover, the hot pressing process can reduce the size of pores, produce small Mg_2_Si particles (from 35 nm to 230 nm) with a homogeneous distribution [14], and increase *J*_c_ from 5 K to 30 K [16]. Moreover, Qu et al. showed that Mg_2_Si is formed at a temperature of 500 °C (hot pressing process) [16]. However, Mg_2_Si and MgB_2_ are created only at 550 °C [16]. Furthermore, there is no reaction between B and Mg SiC during a hot pressing process at 450 °C [16].

The results showed that annealing under high isostatic pressure (HIP) significantly improved the structure of the MgB_2_ material in wires made by using the powder in tube (PIT) method (e.g., small grains, small size, fewer voids, increased connection between grains, increased MgB_2_ density, enhanced homogeneity of MgB_2_ material, and increased density of structural defects) [34,35,36,37,38,39]. Additionally, the HIP process increases the density of pinning centers [35] and mainly increases the density of high-field pinning centers [36], leading to a significant increase in *J*_c_ under a high magnetic field and *B*_irr_ and a decrease in *T*_c_ [35]. Moreover, HIP increases the rate of reaction and accelerates the rate of carbon substitution for boron [36,37,38]. Furthermore, annealing under high isostatic pressure increases the melting point of pure Mg, e.g., 0.1 MPa at 650 °C and 1 GPa at 720 °C [40]. This is very important because it permits heat treatment at higher temperatures for pure Mg in the solid state. Thermal treatments in the solid state will increase the density of MgB_2_ material [35,36,37,38,39].

The aim of this research was to show the impact of large amounts of 10% SiC doping and high isostatic pressure on *J*_c_ at 4.2 K and 20 K and pinning centers. The research shows that the large amount of admixture (10 wt.% SiC) and annealing under high isostatic pressure leads to a significant reduction in the transport critical current density (2 T-100 A/mm^2^) at 20 K. In addition, the research shows that the HIP process allows one to increase the *J*_c_ at 20K in undoped MgB_2_ wires. Moreover, the measurements indicated that large amounts of SiC doping and high isostatic pressure lead to a high *J*_c_ at 4.2 K.

## 2. Materials and Methods

### 2.1. Materials

Multifilament MgB_2_ wires with a Nb barrier, Cu matrix, and Monel sheath were produced at Hyper Tech Research, Inc. in Columbus, OH, USA [41]. These wires were made by using the continuous tube forming and filling (CTFF) method. The in situ MgB_2_ precursor materials were manufactured with Mg and B powders with 99% purity (with a nominal atomic ratio of 1.1:2). The amorphous boron and magnesium grain sizes were ~50 nm and ~40 µm, respectively. The 10 wt.% nano SiC doping MgB_2_ wires had 6 filaments and a diameter of 0.83 mm and 0.63 mm. On the other hand, the undoped MgB_2_ wire had 18 filaments and a diameter of 0.83 mm. The fill factor was approximately 15%, and the wires were sized to either 0.63 mm or 0.83 mm in diameter. The 0.63 mm samples were obtained from further processing of the 0.83 mm diameter wire. Cold drawing of MgB_2_ wire reduced the cross-sectional area of MgB_2_ material by approximately 42%, from 0.81 mm^2^ to 0.46 mm^2^. Both 0.63 mm and 0.83 mm diameter samples were heated together at the same temperature (from 680 °C to 725 °C), pressure (1 GPa), and duration (from 15 min to 25 min)—see Table 1 and Table 2 [42,43]. For the HIP process, the isostatic pressure is first increased, and then the annealing temperature is increased in the second step. After the required time of the HIP process, the temperature was reduced before the isostatic pressure was reduced. All samples had a length of 100 mm. The HIP process was performed by the Institute of High Pressure Physics, PAS, in Warsaw, Poland.

### 2.2. Methods

Critical current (*I*_c_) measurements were made using the four-probe resistive method for a sample of 20 mm length. All samples were measured in a perpendicular magnetic field (Bitter type magnet—14 T) at liquid helium temperature and in accordance with the 1 μV/cm criterion [44,45]. Measurements were made using a DC source in the range from 0 A to 150 A. All critical current measurements carried out using current sweep type—constant magnetic field and increasing current (from 0 A to 150 A by 90 s). The *I*_c_ measurements were conducted by the Institute of Low Temperature and Structure Research, PAS, in Wroclaw, Poland. Furthermore, the *I*_c_ measurements at 10 K, 20 K, and 25 K were made at the Leibniz Institute for Solid State and Materials Research, Dresden, Germany [46,47]. The integrity of the Nb barriers was checked using the field sweep method and temperature sweep method by the Institute of Low Temperature and Structure Research, PAS, in Wroclaw, Poland [44,45]. The *B*_irr_ at 4.2 K was determined from the Kramer model (*B*^0.25^ × *J*_c_^0.5^). On the other hand, *B*_irr_ at 10 K, 20 K, and 25 K were specified from the measurements of the critical current (*I*_c_ = 0 A). Microstructure and composition analyses were performed using an FEI Nova Nano SEM 230 SEM (Hillsboro, OR, USA) by the Institute of Low Temperature and Structure Research, PAS, in Wroclaw, Poland. The structure of the MgB_2_ wires studies were investigated using the secondary electron (SE) method. All samples were embedded in carbon resin and were then polished using standard sandpaper and cleaned in isopropanol.

## 3. Results—Structure

### 3.1. Microstructure of Undoped MgB_2_ Wires

**Figure 1:**
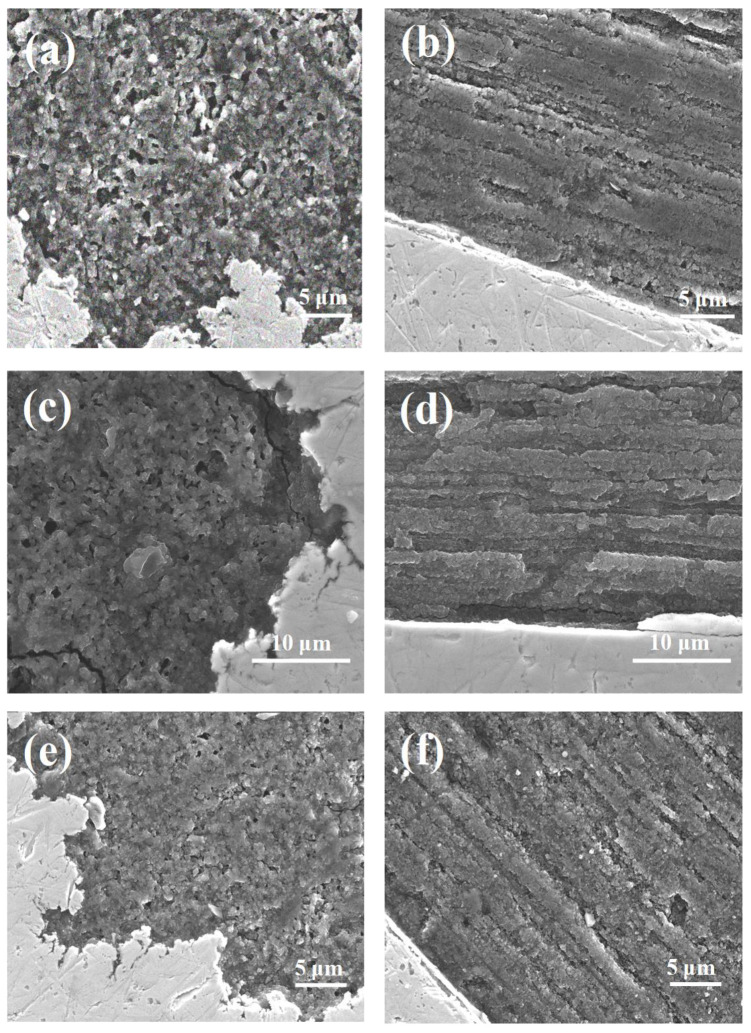
SEM images of undoped MgB_2_ wires with a diameter of 0.83 mm: (**a**) cross-section and (**b**) longitudinal section for sample A (0.1 MPa and 700 °C), (**c**) cross-section and (**d**) longitudinal section for sample B (1 GPa and 700 °C), and (**e**) cross-section and (**f**) longitudinal section for sample C (1 GPa and 725 °C). The metal sheath is the Nb barrier.

The results in Figure 1a,b show that thermal treatment at 700 °C under a low isostatic pressure of 0.1 MPa leads to the presence of a large number of large voids (up to 5 μm) with a non-uniform distribution and to a decrease in the MgB_2_ material density in superconducting wires. The longitudinal section for sample A in Figure 1b is very important because it helps to better investigate the connection between grains. In MgB_2_, the connection between grains significantly influences the transport critical current density properties. Additionally, the SEM images in Figure 1b show that low isostatic pressure annealing creates a thick layer structure (from 2 μm to 3 μm). The studies in Figure 1c,d show that heat treatment under high isostatic pressure increases the density of the MgB_2_ material, reduces the void number by 90%, improves the homogeneity of the superconducting material, and creates a structure with a thinner layer thickness (below 1 μm). This leads to more connections between the grains and more connections between the layers. Further studies show that annealing at 725 °C under an isostatic pressure of 1 GPa leads to the creation of more voids (up to 1 μm) and a structure with thicker layers (up to 2 μm). This reduces the number of connections between the grains and layers and reduces the density and uniformity of the MgB_2_ material in the wire (Figure 1e,f). The tests conducted for sample D show that the longer annealing times at 700 °C and 1 GPa create a superconducting material structure similar to the structure of sample B.

Jung et al. [6] showed that the MgB_2_ material shrinks by 25% during the formation of the superconducting phase. This leads to a significant reduction in the density and uniformity of the superconducting material and intergrain connections. This reduces the transport critical current density in MgB_2_ wires. Currently, voids are the biggest problem in MgB_2_ materials and limits the application possibilities. Our results show that this problem can be solved by using annealing under high isostatic pressure (1 GPa) in the solid state of Mg. This process leads to a significant reduction in the number of voids. Additionally, our research indicates that thermal treatment under high isostatic pressure and the liquid state of Mg slightly reduce the number of voids in the MgB_2_ superconducting material.

### 3.2. Microstructure of SiC Doped MgB_2_ Wires

**Figure 2:**
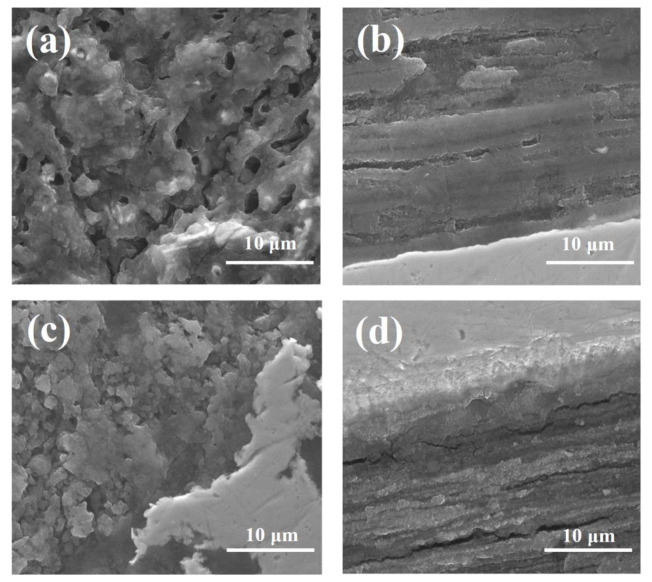
SEM images of 10% SiC-doped MgB_2_ wires with a 0.83 mm diameter: (**a**) cross-section and (**b**) longitudinal section for sample E (0.1 MPa and 700 °C) and (**c**) cross-section and (**d**) longitudinal section for sample F (1 GPa and 700 °C). The metal sheath is the Nb barrier.

The results in Figure 2a show that annealing at low pressure creates a larger and greater number of voids (up to 10 μm), leading to a reduction in the connection between grains. Moreover, these voids are heterogeneously distributed in the structure of the MgB_2_ material. Previous research has shown that low pressure forms an inhomogeneous distribution of Si particles [37].

**Figure 3:**
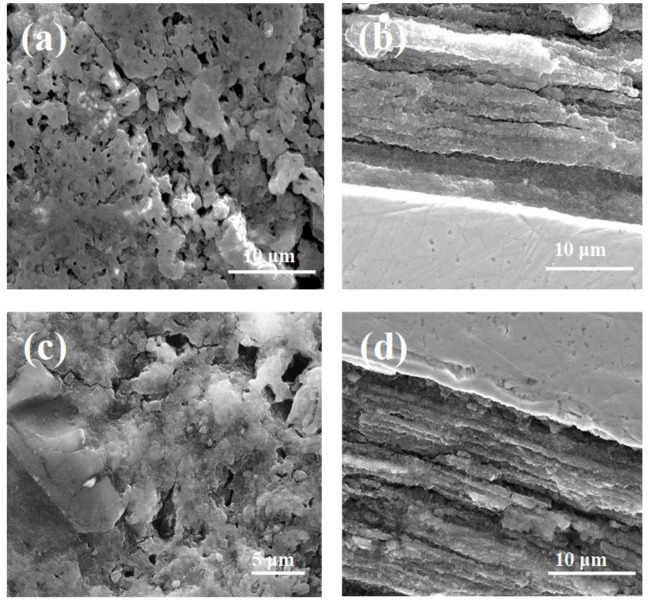
SEM images of 10% SiC MgB_2_ wires with a 0.83 mm diameter: (**a**) cross-section and (**b**) longitudinal section for sample H (1 GPa, 725 °C, 15 min) and (**c**) cross-section and (**d**) longitudinal section for sample I (1 GPa, 700 °C, 25 min). The metal sheath is the Nb barrier.

The SEM image in Figure 2b indicates that low pressure creates large and long voids in a lamellar structure with thick layers (up to 5 μm). The results in [37] showed that Si particles are located outside and between the lamellar structure. This may be due to the non-substitution of Si into the crystal lattice [10,18,21]. This also leads to reduced connections between grains. Li et al. [20] and Zhang et al. [22] indicated that impurities weaken intergrain connections. In addition, Serrano et al. suggested that a SiC admixture weakly increases the number of connections between grains. This may suggest that the connections between grains are created mainly by Mg grains. On this basis, we can deduce that Mg grains mainly influence the efficiency of connections between grains. Furthermore, the investigation indicated that annealing under a high isostatic pressure of 1 GPa decreased the size by less than 1 μm and the number (by 90%) of voids, increased the number of connections between grains, created more connections between grains, created a lamellar structure with thin layers (Figure 2c,d), produced smaller Si particles [37], and produced a more homogeneous distribution of Si particles [37]. The SEM images in Figure 3a,b show that a higher annealing temperature (725 °C) and high isostatic pressure of 1 GPa form larger (up to 5 μm), longer, and an increased number of voids; create a lamellar structure with thick layers (up to 5 μm); and decrease the number of connections between grains. Furthermore, the SEM images in Figure 3c,d indicate that a longer annealing time at 700 °C and a high isostatic pressure create longer, larger (up to 3 μm), and an increased number of voids; decrease the density of the MgB_2_ material; and decrease the connections between grains compared to sample F.

However, the longer annealing time produces a higher density structure compared to sample H, which was heated at a higher temperature. The investigation showed that pure Mg at 1 GPa transitions from the solid state to the liquid state at approximately 720 °C [40]. These results indicate that high isostatic pressure increases the effective density of the MgB_2_ material during the reaction in solid-state Mg. Moreover, the results indicate that annealing under high isostatic pressure in the liquid state of Mg produces a similar structure to sample E (annealing under low pressure).

**Figure 4:**
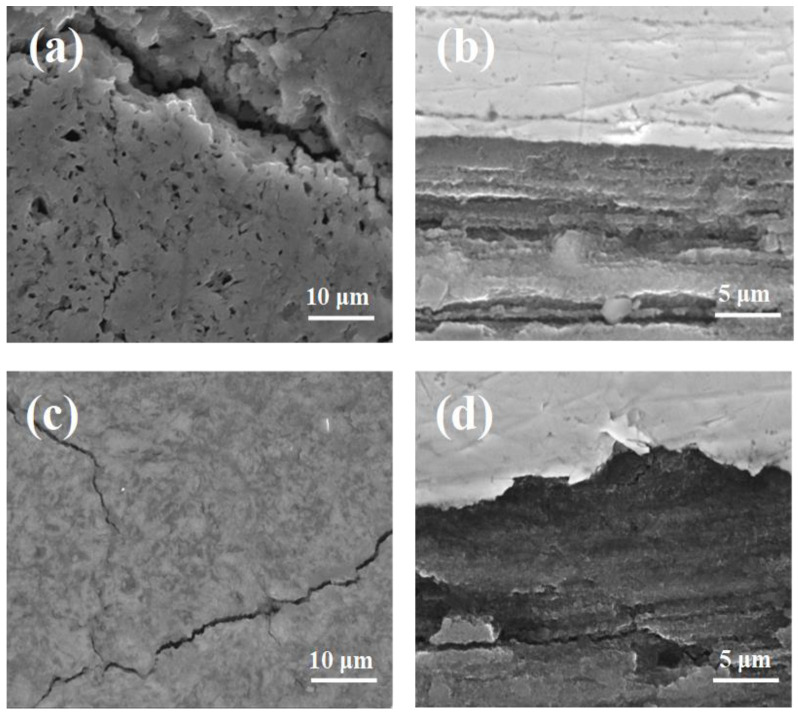
SEM images of 10% SiC-doped MgB_2_ wires with a 0.63 mm diameter: (**a**) cross-section and (**b**) longitudinal section for sample J (0.1 MPa and 700 °C) and (**c**) cross-section and (**d**) longitudinal section for sample K (1 GPa and 700 °C). The metal sheath is the Nb barrier.

**Figure 5:**
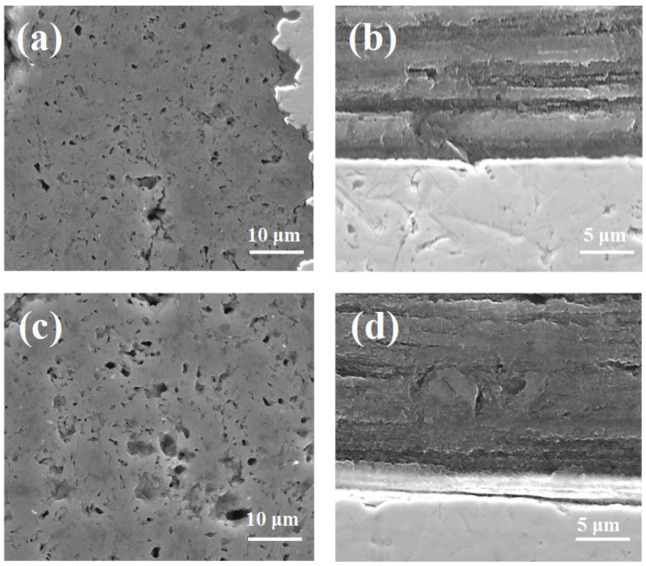
SEM images of 10% SiC-doped MgB_2_ wires of 0.63 mm diameter: (**a**) cross-section and (**b**) longitudinal section for sample L (1 GPa, 725 °C, 15 min) and (**c**) cross-section and (**d**) longitudinal section for sample M (1 GPa, 700 °C, 25 min). The metal sheath is the Nb barrier.

The results in Figure 4a,b show that annealing under low isostatic pressure forms long, large (up to 5 μm) and more numerous voids; reduces the connection between grains; and creates a lamellar structure with thick layers (up to 5 μm). This in turn reduces the density and homogeneity of the MgB_2_ material. The SEM images in Figure 4c,d indicate that annealing under high isostatic pressure significantly reduces the number, size and length of voids; increases the connections between grains; and decreases the thickness of layers, increasing the density and homogeneity of the MgB_2_ material. A higher annealing temperature with high isostatic pressure forms long, large (up to 5 μm), and more numerous voids; decreases the connections between grains; and creates a lamellar structure with thick layers (Figure 5a,b), decreasing the density and homogeneity of the MgB_2_ material. Moreover, the SEM images in Figure 5c,d show that a long annealing time and high isostatic pressure create a small number of large voids (from 1μm to 3μm) and voids with a short length, form a lamellar structure with thin layers (up to 2μm), and slightly reduce the connections between grains compared to sample K. These temperature and pressure parameters only slightly decreased the density and homogeneity of the MgB_2_ material for sample M.

### 3.3. Comparison of the Structure of Undoped and SiC-Doped MgB_2_ Wires

Cold drawing of in situ MgB_2_ wires causes the Mg grains to elongate [48]. This means that the 0.63 mm wires contain smaller Mg grains than the 0.83 mm wires. The SEM images in Figure 2a,b and Figure 4a,b show that sample J (0.63 mm, 0.1 MPa) has longer, larger, and an increased number of voids than sample E (0.83 mm, 0.1 MPa), indicating that smaller Mg grains react faster than large Mg grains at 700 °C (Mg was in the liquid state). The SEM images in Figure 2c,d and Figure 4c,d indicate that the MgB_2_ material density is higher in sample K (0.63 mm, 1 GPa) than in sample F, indicating that high isostatic pressure increases the MgB_2_ density with smaller Mg grains and a smaller wire diameter (reaction in the solid-state Mg). Moreover, these results suggest that the size of Mg grains does not influence the rate of reaction for the solid-state Mg. Furthermore, the results show that sample L (0.63 mm, 1 GPa, 725 °C) has larger, longer, and an increased number of voids than sample H (0.83 mm, 1 GPa, 725 °C). This indicates that smaller Mg increases the rate of reaction for liquid-state Mg (Figure 2a,b and Figure 4a,b). Furthermore, these results indicate that high isostatic pressure does not increase the density of MgB_2_ material in liquid-state Mg (smaller diameter and smaller Mg grains). Additionally, the results in Figure 3c,d and Figure 5c,d show that a long annealing time, high isostatic pressure, reaction in the solid-state Mg, and smaller Mg grains increase the density of MgB_2_ material in wires. Shcherbakova et al. [27] indicated that MgB_2_ grains grow faster in the c direction than in the ab direction. This also leads to the formation of a lamellar structure in MgB_2_ wires. Studies have shown that the lamellar structure significantly increases *J*_c_ and *B*_irr_ and improves the connections between grains [39]. Our results showed that thinner and longer Mg grains obtain a lamellar structure with thinner layers and improve the longitudinal connection between grains. The research performed for undoped MgB_2_ wires shows that the HIP process yields a higher MgB_2_ material density and creates a greater number of connections between grains in smaller diameter wires (d = 0.63 mm [49]) than for wires with a larger diameter of 0.83 mm (Figure 1). Additionally, the results show that the diameter of the undoped MgB_2_ wire barely influences the structure of the superconducting material for the synthesis reaction in the Mg liquid state (Figure 1 and [49]).

## 4. Results—Transport Measurements

### 4.1. Analysis of the Critical Current Density and Pinning Centers for Undoped MgB_2_ Wires with the Diameter of 0.83 mm

**Figure 6:**
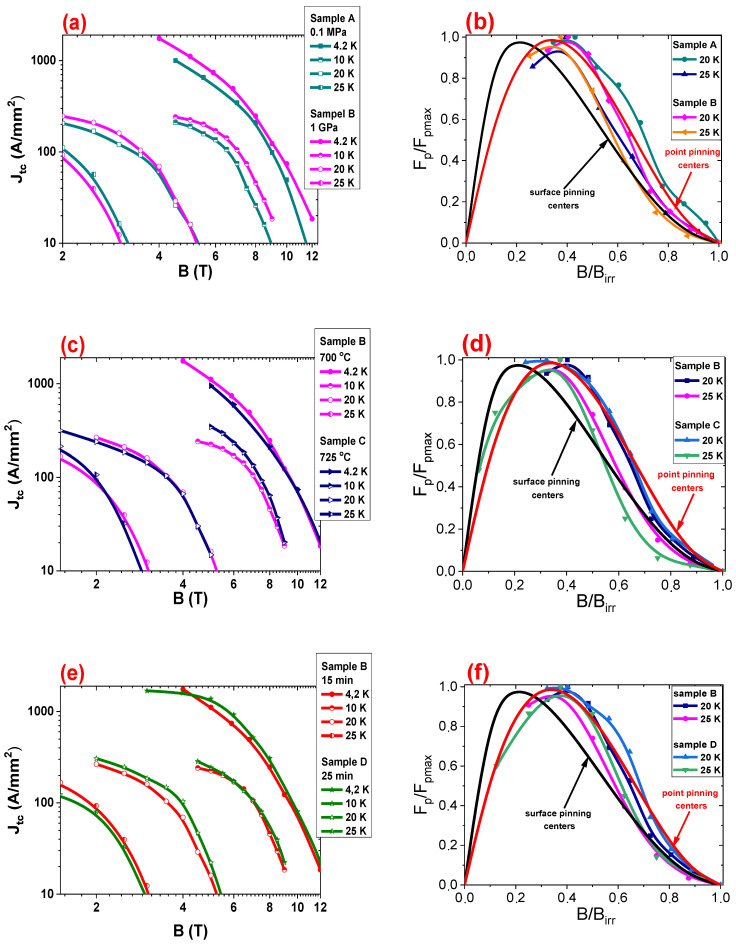
(**a**,**c**,**e**) The transport critical current density (*J*_tc_) as a function of the perpendicular magnetic field (*B*) at 4.2 K, 10 K, and 20 K for undoped MgB_2_ wires with a 0.83 mm diameter. (**b**,**d**,**f**) The reduced pinning force depending on the reduced magnetic fields for undoped MgB_2_ wires.

The critical current density in MgB_2_ wires depends on two factors: the density of pinning centers and the connection between grains. Different types of pinning centers can effectively trap lattice vortices in various ranges of magnetic fields [7,49,50]. Better understanding and explanations of the factors that influence the critical current density [7,50] will be useful for improving superconducting wires for commercial applications. The results in Figure 6a at 4.2 K and 10 K show that thermal treatment under a high isostatic pressure of 1 GPa will increase *J*_tc_ by 35% in all magnetic fields. These results indicate that the 1 GPa isostatic pressure at 4.2 K and 10 K increase the density of low, middle, and high-field pinning centers (low-field pinning centers to anchor vortex lattice in low magnetic fields, middle-field pinning centers to trap vortex lattice in middle magnetic fields, and high-field pinning centers to anchor vortex lattice in high magnetic fields). Further studies at 20 K show that the HIP process improves *J*_tc_ by 30% in magnetic fields from 0 T to 4 T. Currently, the Dew–Hughes model is the most used for the study of pinning centers in superconducting wires [51]. This model demonstrates the dominant pinning mechanism. The dominant pinning mechanism indicates the type of pinning centers that are the most abundant in the superconducting material. This allows one to identify structural defects, which in turn allows one to achieve the highest transport critical current density in superconducting wires and tape. The results in Figure 6b indicate that heat treatment under a high isostatic pressure of 1 GPa does not change the dominant pinning mechanism at 20 K. This indicates that the isostatic pressure of 1 GPa at 20 K increases the density of the low- and middle-field pinning centers and slightly increases the density of the high-field pinning centers. Additionally, the results (Figure 6a,b) show that high isostatic pressure annealing poorly increases *J*_tc_ at 25 K and does not change the dominant pinning mechanism. These results indicate that the HIP process slightly increase the density and improves the pinning centers at 25 K. The results in Figure 6c,d show that higher annealing temperatures and a high isostatic pressure of 1 GPa slightly increase *J*_tc_ in the range of 4.2 K to 25 K and does not change the dominant pinning mechanism at 20 K and 25 K. The measurement results in Figure 6e indicate that a longer annealing time and an isostatic pressure of 1 GPa slightly increase *J*_tc_ in the temperature range from 4.2 K to 25 K. In addition, the Dew–Hughes model [51] shows that a longer annealing time under an isostatic pressure of 1 GPa does not lead to changes of the dominant pinning mechanism at 20 K and 25 K (Figure 6f). These results show that increasing the annealing temperature by 25 °C with an annealing time of 10 min under an isostatic pressure of 1 GPa slightly increases the pinning center density in all regimes (low, middle, and high magnetic fields).

The results presented in [51] (undoped MgB_2_ wires with a diameter of 0.63 mm) and Figure 6a (undoped MgB_2_ wires with a diameter of 0.83 mm) show that thermal treatment under high isostatic pressure significantly increases *J*_tc_ at 20 K in undoped MgB_2_ wires with a diameter of 0.63 mm. The measurements shown in [51] (undoped MgB_2_ wires with a diameter of 0.63 mm) and Figure 6a (undoped MgB_2_ wires with a diameter of 0.83 mm) indicate that thermal treatment under high and low isostatic pressure increases *J*_tc_ at 4.2 K in undoped MgB_2_ wires with a diameter of 0.83 mm. The SEM images in [52] and [53] show that 0.63 mm wires after the HIP process have a higher density of undoped MgB_2_ material than 0.83 mm wires. The SEM images in [52] and [53] indicate that 0.63 mm wires after annealing at low isostatic pressure have a slightly lower density for undoped MgB_2_ wires with a diameter of 0.83 mm. These results indicate that Mg grains with a longer length and smaller diameter create better connections between grains and more effective pinning centers at 20 K. In addition, the reaction of solid-state Mg (pressure 1 GPa) produces a higher density of low- and middle-field pinning centers. The large Mg grains with a smaller length create better connections and a higher density of pinning centers at 4.2 K. The Dew–Hughes analysis [51] shows that the high isostatic pressure, wire diameter, annealing time, and annealing temperature did not have an influence on the dominant pinning mechanism in undoped MgB_2_ wires.

### 4.2. Analysis of the Critical Current Density and Pinning Centers for SiC-Doped MgB_2_ Wires with a Diameter of 0.83 mm

**Figure 7:**
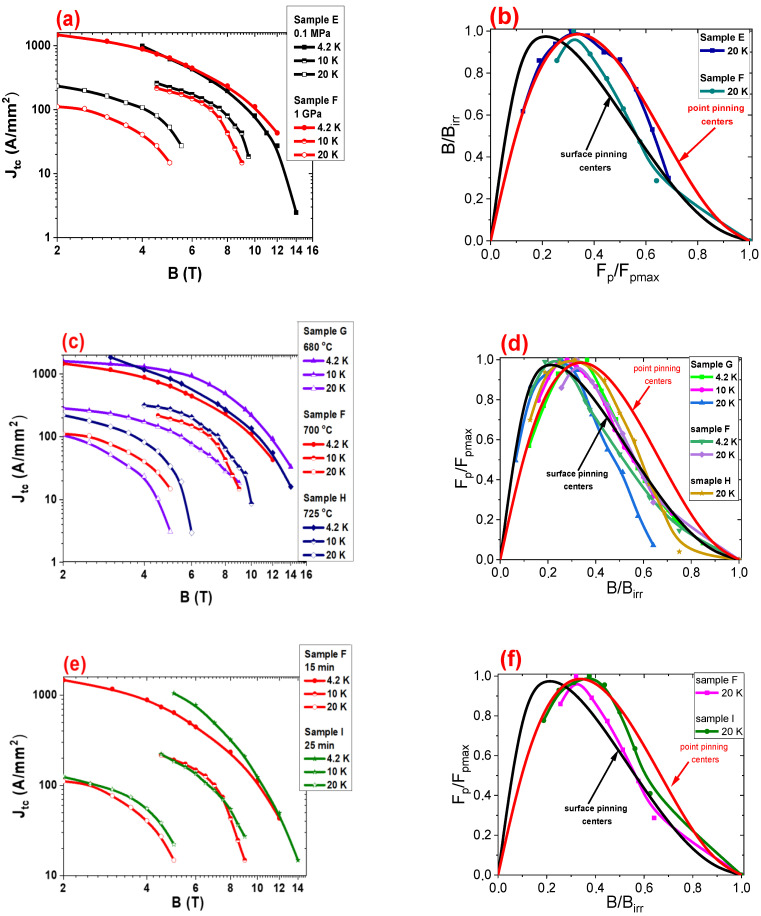
(**a**,**c**,**e**) The transport critical current density (*J*_tc_) as a function of the perpendicular magnetic field (*B*) at 4.2 K, 10 K, and 20 K for SiC doped MgB_2_ wires with a 0.83 mm diameter. (**b**,**d**,**f**) The reduced pinning force depending on the reduced magnetic fields for SiC doped MgB_2_ wires.

The results in Figure 7a show that annealing under high isostatic conditions increases *J*_tc_ at 4.2 K in high magnetic fields (above 8 T) but decreases *J*_tc_ at 4.2 K in low magnetic fields. High isostatic pressure increases the density of high-field pinning centers due to the increasing dislocations, rate of substitution of C for B, strain, and precipitation inside grains [7]. Moreover, annealing with high isostatic pressure decreases the density of low-field pinning centers (surface pinning centers, e.g., voids [49]). The reduction in voids is caused by the increase in MgB_2_ material density (Figure 2). Figure 7a shows that annealing under high isostatic pressure slightly decreases *J*_tc_ by 8% at 10 K. Further measurements indicate that thermal treatment under high isostatic pressure significantly decreases *J*_tc_ by a factor of three in low, middle, and high magnetic fields at 20 K (Figure 7a). The transport measurements showed that the HIP process increased *J*_tc_ at 20 K in undoped MgB_2_ wires (Figure 6a). Further studies have shown that SiC doping and thermal treatment under low isostatic pressure (sample E—Figure 7a) increases *J*_tc_ in the middle and high magnetic fields at 20 K compared to an undoped MgB_2_ wire (sample A). This indicates that a large reduction in *J*_tc_ at 20 K in sample F is the result of the HIP process and a large amount of SiC additive. However, the SEM images show that sample F contains more connections between grains than sample E. The analysis of pinning mechanisms [7,50,53] indicates that samples E and F have the same dominant pinning mechanism at 10 K and 20 K (Figure 7b). In addition, increasing the temperature from 4.2 K to 20 K does not remove the structural defects in the MgB_2_ material, which creates pinning centers. Increasing the temperature can only lead to the clustering of several pinning centers into one pinning center. Moreover, increasing the temperature from 4.2 K to 20 K only slightly increases the coherence length [49,54]. This indicates that increasing the temperature does not affect the parameters and efficiency of the pinning centers (e.g., precipitation and impurities). Si particles accumulate at the grain boundaries [5] because Si particles do not substitute in the critical lattice [10,18,21]. Moreover, the Si particles located on the grain boundaries might create dislocations and strains in the MgB_2_ grains and connections [19]. Serquis et al. [32] stated that the HIP process creates dislocations in the MgB_2_ grains and connections, indicating that SiC doping and the HIP process mainly form pinning centers in the connections between grains and on the grain boundaries. Furthermore, Kazakov et al. [31] and Dou et al. [26] showed that strains (e.g., substitution of C for B) led to a local reduction in *T*_c_. These results indicate that the significant reduction in *J*_tc_ as a result of increasing the temperature from 4.2 K to 20 K is the result of weakening the connections between MgB_2_ grains. The connections worsened due to the clustering of structural defects. Zhang et al. [22] and Li et al. [20] indicated that precipitation led to the weakening of connections between grains. The second reason for the reduction of *J*_tc_ at 20 K in sample F may be the state of Mg during the synthesis reaction. Research shows that the synthesis reaction in sample E was liquid-state Mg [40]. However, the synthesis reaction in sample F was solid-state Mg [40]. This indicates that liquid-state Mg creates more effective connections between grains than solid-state Mg at 20 K for MgB_2_ wires with a large amount of SiC impurities.

Figure 7b shows that annealing at 680 °C under a high isostatic pressure of 1 GPa significantly increases *J*_tc_ in middle and high magnetic fields but decreases *J*_tc_ in low magnetic fields at 4.2 K compared to samples F and H. Moreover, these results indicate that annealing at higher temperature (725 °C) leads to increasing *J*_tc_ in low magnetic fields at 4.2 K. Furthermore, the investigation showed that annealing at 680 °C under an isostatic pressure of 1 GPa leads to a significant decrease in *J*_tc_ at 10 K and 20 K compared with *J*_tc_ in samples F and H. However, for sample H (725 °C and 1 GPa), the higher annealing temperature slightly increases *J*_tc_ at 10 K and significantly increases *J*_tc_ at 20 K compared with *J*_tc_ in sample F.

The calculation of the dominant pinning mechanism by using the Dew–Hughes model [51] shows that sample G (1 GPa and 680 °C) has a dominant point pinning mechanism between 4.2 K to 10 K (from 0 to 0.35—Figure 7d) and a dominant surface pinning mechanism (from 0 to 0.35—Figure 7d) at 20 K. Figure 7d shows that the longer annealing time (sample H) and the high isostatic pressure of 1 GPa does not change the dominant pinning mechanism at 20 K. The results for sample H suggest that *J*_tc_ in SiC-doped MgB_2_ wires mainly depends on the density and type of pinning centers and does not depend on the connections between grains because sample H has fewer connections between grains compared to samples F and G. Based on [37], sample G is an MgB_2_ material with high homogeneity and high density, leading to a large number of connections between the grains. Increasing the temperature from 4.2 K to 20 K does not break the connections between the grains. This indicates that a significant reduction in *J*_tc_, similar to sample F, is not related to the destruction of intergrain connections but only to the decrease in the density of pinning centers (the clustering of pinning centers) or a change in the dominant pinning mechanism (e.g., dominant point pinning mechanism at 4.2 K to the dominant surface pinning mechanism (20 K)—Figure 7d). Additionally, the research shows that Si particles do not substitute into the crystal lattice [10,18,21]. This indicates that Si particles are on grain boundaries and connections between grains. Additionally, annealing under an isostatic pressure of 1 GPa creates structural defects [34]. This indicates that the HIP process and 10% SiC doping create a large number of structural defects at grain boundaries and connections between grains (similar to sample F). The clustering of pinning centers, the high density of pinning centers, and the change in the dominant pinning mechanism may lead to a weakening of the connections between grains at 20 K. Furthermore, a low annealing temperature (680 °C), 10% SiC doping, and reaction in solid-state Mg might create a weak connection between grains at 20 K.

The results in Figure 7e show that a longer annealing time and high isostatic pressure significantly increase *J*_tc_ at 4.2 K in low and middle magnetic fields. Additionally, a longer annealing time and high isostatic pressure appear to not change *J*_tc_ at 10 K and 20 K. Moreover, the Dew–Hughes model [51] indicated that a longer annealing time and the isostatic pressure of 1 GPa does not significantly change the dominant pinning mechanism at 20 K (Figure 7f). However, a longer annealing time and high isostatic pressure only slightly increase the density of high-field pinning centers. The SEM images showed that the density of MgB_2_ and the size and number of voids in samples F and I are similar, indicating that the substitution of C for B during reaction in solid-state Mg requires a longer annealing time (more than 25 min).

Based on the Dew–Hughes model [51], it can be indicated that a significant reduction in *J*_tc_ at 20K is caused by the change of the dominant pinning mechanism from point to surface and a decrease of the density of pinning centers.

### 4.3. Analysis of the Critical Current Density and Pinning Centers for SiC-Doped MgB_2_ Wires with a Diameter of 0.63 mm

**Figure 8:**
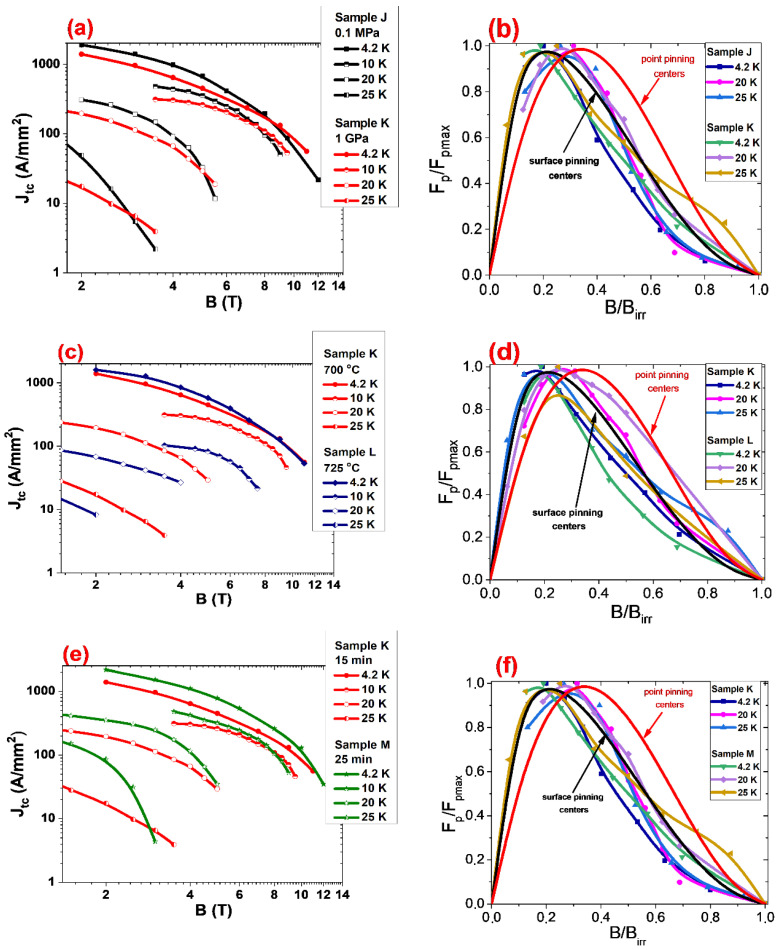
(**a**,**c**,**e**) The transport critical current density (*J*_tc_) as a function of the perpendicular magnetic field (*B*) at 4.2 K, 10 K, and 20 K for SiC doped MgB_2_ wires with a 0.63 mm diameter. (**b**,**d**,**f**) The reduced pinning force depending on the reduced magnetic fields for SiC doped MgB_2_ wires.

The results in Figure 8a (0.63 mm diameter) show that annealing under a high isostatic pressure of 1 GPa increases *J*_tc_ in high magnetic fields and decreases *J*_tc_ in low and middle magnetic fields by between 4.2 K and 25 K. The analysis of the pinning centers (Figure 8b) by using the Dew–Hughes model [51] shows that thermal treatment under high isostatic pressure of 1 GPa does not change the dominant pinning mechanism in a range from 4.2 K to 25 K. This indicates that the reduction of *J*_tc_ (Figure 8a) in low and middle magnetic fields is mainly due to the reduction of the pinning center density. In addition, the Dew–Hughes model [51] indicated the appearance of the dominant point pinning mechanism in a range from 0.75 to 1. This leads to the increase of *J*_tc_ in the high magnetic fields. The SEM images in Figure 4 show that sample K has a much higher density of superconducting material than sample J. These results indicate that a higher density of MgB_2_ material might decrease the density of surface and point pinning centers.

The results in Figure 8c (0.63 mm diameter) show that a higher annealing temperature and higher isostatic pressure slightly increase *J*_tc_ at 4.2 K and significantly decrease *J*_tc_ by between 10 K and 25 K. The pinning center analysis indicates that a higher annealing temperature and higher isostatic pressure slightly increase the density of pinning centers and does not change the dominant pinning mechanism at 4.2 K (Figure 8d). From 10 K to 25 K, the higher annealing temperature and high isostatic pressure decrease the density of each type of pinning center. The SEM images in Figure 4a,b and Figure 5a,b show that sample J (0.1 MPa, 700 °C) and sample L (1 GPa, 725 °C) have a similar number of connections between grains. The Dew–Hughes analysis [51] indicates that the reduction of *J*_tc_ at 20 K and 25 K is mainly caused by the reduction of the pinning center density (Figure 8d), because the dominant pinning mechanism does not change significantly at 20 K and 25 K.

The results in Figure 8e (0.63 mm diameter) show that a longer annealing time and high isostatic pressure significantly increase *J*_tc_ in low and middle magnetic fields between 4.2 K and 25 K and decrease *J*_tc_ in high magnetic fields in the same temperature range. The Dew–Hughes model shows that the longer annealing time and 1 GPa isostatic pressure does not lead to changes of the dominant pinning mechanism at 4.2 K, 20 K, and 25 K (Figure 8f). This indicates that the longer annealing time and HIP process allows one to increase the pinning center density. The SEM images in Figure 4c,d and Figure 5c,d show that sample K has more connections between grains than sample M. These results indicate that *J*_tc_ in sample M is dependent on pinning centers rather than the connection between grains. Furthermore, the SEM images (Figure 5c,d) indicate that voids, Mg_2_Si particles, and Si particles can create low and middle pinning centers [49].

### 4.4. Comparison of the Results of J_tc_ and Pinning Mechanisms for Undoped and 10 wt.% MgB_2_ Wires

Earlier studies have shown that MgB_2_ wires with a large amount of SiC admixture should be fabricated with excess Mg or by maximizing the amount of MgB_2_ superconducting phase [14,18,20,21,22]. Studies conducted by Li et al. showed that the Mg_2_Si phase is formed first, followed by the MgB_2_ phase and free Si in SiC-doped MgB_2_ superconductors [8]. In addition, studies have shown that hot pressing (HP) accelerates the formation of the Mg_2_Si phase (500 °C [16]). This indicates that the heat treatment under isostatic pressure accelerates the formation of the MgB_2_ phase. Images from a scanning electron microscope with a backscattering electron (BSE) function do not show pure Mg.

The results presented by Susner et al. [48] showed that cold drawing of unreacted MgB_2_ wires reduced the thickness and increased the length of Mg grains. The results for sample E (0.1 MPa, 0.83 mm diameter) and sample J (0.1 MPa, 0.63 mm diameter) indicate that *J*_tc_ in the temperature range from 4.2 K to 20 K is similar. *J*_tc_ is similar in undoped MgB_2_ wires with diameters of 0.63 mm and 0.83 mm after heat treatment at low isostatic pressure. This indicates that the size and length of Mg grains during reaction in the liquid state of Mg does not influence *J*_tc_ or the different types of pinning centers in SiC-doped and undoped MgB_2_ wires. Furthermore, for annealing sample F (0.83 mm) and sample K (0.63 mm) under high isostatic pressure, the smaller Mg grains produced in the reaction in solid-state Mg does not increase *J*_tc_ at 4.2 K but significantly increases *J*_tc_ at 10 K and 20 K. On the basis of the SEM images of samples F and K, one might deduce that the higher density of the MgB_2_ material and more connections between grains increase the density of low- and middle-field pinning centers and significantly increases the density of high-field pinning centers. The measurements for undoped wires (sample B—0.83 mm and [52]—d = 0.63 mm) show that the smaller wire diameter and HIP process significantly increases *J*_tc_ in low and medium magnetic fields at 20 K but does not improve *J*_tc_ in high magnetic fields at 20 K. In addition, the investigation for sample H (0.83 mm, 725 °C) and sample L (0.63 mm, 725 °C) showed that a higher annealing temperature, high isostatic pressure, smaller Mg grains, and a reaction in the liquid state of Mg had no influence on *J*_tc_ at 4.2 K but significantly decreased *J*_tc_ at 10 K and 20 K for both samples because the structures of samples H and L are similar. These results suggest that *J*_tc_ is mainly dependent on the density of pinning centers. The low-field pinning centers do not act in high magnetic fields [49] and high temperatures. The measurements indicate that a higher annealing temperature (725 °C), HIP process, and smaller wire diameter do not increase *J*_tc_ at 4.2 K and 20 K in undoped MgB_2_ wires. However, a very high annealing temperature ([52]—740 °C) leads to a significant reduction in *J*_tc_ at 20 K in undoped MgB_2_ wires. The results for sample I (0.83 mm) and sample M (0.63 mm) showed that a longer annealing time, high isostatic pressure, smaller Mg grains, and reaction in solid-state Mg slightly increased *J*_tc_ at 4.2 K and significantly increase *J*_tc_ at 10 K and 20 K. The SEM images showed that sample M has a higher density of MgB_2_ material than sample I. These results indicate that a higher density of MgB_2_ material and more connections between grains produces a high density of pinning centers.

Comparing the results of SiC-doped and undoped [52] MgB_2_ 0.63 mm wires after heat treatment (700 °C for 15 min) at low isostatic pressure shows that doping increases *J*_tc_ in high magnetic fields (e.g., SiC doped at 4.2 K, 100 A/mm^2^ in 10 T and undoped at 4.2 K, 100 A/mm^2^ at 8 T [51]) and slightly decreases *J*_tc_ in low magnetic fields between 4.2 K and 20 K. SEM images show that the structures of doped and undoped samples are similar (grain size and number of connections between grains), indicating that the SiC dopant creates high-field pinning centers and reduces the density of low-field pinning centers. Similar results were obtained for SiC-doped and undoped MgB_2_ wires sized 0.83 mm (Figure 6a and Figure 7a).

Comparing the results for doped and undoped [52] MgB_2_ 0.63 mm wires after heat treatment at 725 °C under high isostatic pressure shows that there is a significant reduction in *J*_tc_ between 10 K and 20 K in MgB_2_-doped wire. These samples have a similar structure according to the SEM images, suggesting that, in both doped and undoped [51] MgB_2_ wires, the reaction in liquid-state Mg is mainly dependent on the density of pinning centers and much less dependent on intragrain connections. Figure 6e and Figure 7e indicate that a longer annealing time under high isostatic pressure slightly increases *J*_tc_ in doped and undoped MgB_2_ 0.83 mm wires.

On the basis of the Dew–Hughes model [51], it can be indicated that the large amount of SiC doping creates the greater number of low-field pinning centers [49] at 20 K than undoped MgB_2_ material. Additionally, the Dew–Hughes analysis [51] shows that wires with a 0.63 mm diameter and a large amount of dopant have more low-field pinning centers than doped MgB2 wires with 0.83 mm diameter at 20 K.

Kim et al. [55] indicated that amorphous boron and excess Mg yield higher *J*_tc_ than crystalline boron. Comparing MgB_2_ wires with a 10% SiC admixture (amorphous boron, Mg_1.1_B_2_) and MgB_2_ wires with a 2% C admixture (crystalline boron, MgB_2_ [36]), the *J*_tc_ of the SiC-doped sample is higher than the *J*_tc_ in 2% C-doped MgB_2_ wires between 10 K and 25 K after heat treatment at low isostatic pressure. Further results indicate that heat treatment under high isostatic pressure significantly increases *J*_tc_ in 2% C-doped MgB_2_ wires (crystalline boron) between 10 K and 25 K compared to SiC-doped and undoped MgB_2_ wires [51]. These results show that too much dopant and too many structural defects (HIP process, cooling, without additives) lead to a reduction in *J*_tc_ at high temperatures.

## 5. Conclusions

The combination of 10 wt.% SiC doping and low isostatic pressure increases the density of high-field pinning centers and reduces the density of low- and middle-pinning centers between 4.2 K and 25 K. The SiC doping and high isostatic pressure significantly reduce the density of low- and middle-field pinning centers and significantly increase the density of high-field pinning centers in wires with a diameter of 0.63 mm between 4.2 K and 25 K. The efficiency of high isostatic pressure applied to 0.83 mm wires is much lower than that applied to 0.63 mm wires.

Mg grains with a smaller diameter and longer length correspond to higher *J*_tc_ at 20 K compared to SiC-doped and undoped wires with Mg grains with a larger diameter and shorter length. This indicates that longer Mg grains with smaller thicknesses create better connections between the grains and more efficient pinning centers at 20 K. Large grains with a shorter length create better connections between grains and more pinning centers at 4.2 K. This may be related to the lower density of MgB_2_ material with a 0.83 mm diameter (more voids that form both low- and middle-fields pinning centers). A larger number of voids at 20 K may lead to the weakening of the connections between grains because several voids and weak areas of superconductivity are combined into one large non-superconducting area.

The large amount of 10 wt.% SiC admixture decreases *J*_tc_ at 20 K in both 0.63 mm and 0.83 mm wires after the HIP process. This may be associated with too many structural defects at grain boundaries and connections between grains. These defects at 4.2 K create a high density of different types of pinning centers. Increasing the temperature from 4.2 K to 20 K causes the clustering of several structural defects into one structural defect (transition of pinning centers, e.g., from dominant point pinning mechanism to dominant surface pinning mechanism), leads to a decrease in the density of the pinning centers, and weakens the connections between grains at 20 K.

The *J*_tc_ in SiC-doped and undoped MgB_2_ wires after reaction in the liquid state of Mg is mainly dependent on the density of the pinning centers. High isostatic pressure during the liquid-state reaction does not increase the density of MgB_2_ material in 0.83 mm and 0.63 mm wires. The J_tc_ in SiC-doped and undoped wires after the Mg solid-state reaction is mainly dependent on the connections between the grains and the pinning centers. This is especially evident at 20 K as SEM studies show that high isostatic pressure increases the density of MgB_2_ material more strongly in SiC-doped and undoped wires with a smaller diameter (0.63 mm) than in wires with a larger diameter (0.83 mm).

Our research shows that MgB_2_ wires used in devices operating at the temperature of liquid helium can be designed with a large amount of admixture and can be thermally treated under high isostatic pressure for optimal superconductivity properties. However, MgB_2_ wires used in devices operating at 20 K must have a small amount of admixture when heated under high isostatic pressure.

## Figures and Tables

**Table 1:** Heat treatment parameters of the undoped MgB_2_ superconductor wires.

No.	Annealing Temperature (°C)	Annealing Time (min)	Isostatic Pressure	Wire Diameter (mm)	*B*_irr_ (4.2 K) T	*B*_irr_ (10 K) T	*B*_irr_ (20 K) T	*B*_irr_ (25 K) T
A	700	15	0.1 MPa	0.83	14.5	10	5.8	3.8
B	700	15	1 GPa	0.83	15	11	6.2	4
C	725	15	1 GPa	0.83	15	11	6.2	4
D	700	25	1 GPa	0.83	15	11	6.2	4

**Table 2:** Heat treatment parameters of the 10% SiC doped MgB_2_ superconductor wires.

No.	Annealing Temperature (°C)	Annealing Time (min)	Isostatic Pressure	Wire Diameter (mm)	*B*_irr_ (4.2 K)T	*B*_irr_ (10 K) T	*B*_irr_ (20 K) T	*B*_irr_ (25 K) T
E	700	15	0.1 MPa	0.83	15.2	11	8	
F	700	15	1 GPa	0.83	16	12	7.8	
G	680	15	1 GPa	0.83	16.5	12	7.8	
H	725	15	1 GPa	0.83	16	12	8	
I	700	25	1 GPa	0.83	16	12	8	
J	700	15	0.1 MPa	0.63	15	11	8	3.8
K	700	15	1 GPa	0.63	16	12	8	4
L	725	15	1 GPa	0.63	16	12	8	4
M	700	25	1 GPa	0.63	16	12	8	4

## Data Availability

The data presented in this study are available on reasonable request from the corresponding authors.
